# Mutation Linked to Autosomal Dominant Nocturnal Frontal Lobe Epilepsy Reduces Low-Sensitivity α4β2, and Increases α5α4β2, Nicotinic Receptor Surface Expression

**DOI:** 10.1371/journal.pone.0158032

**Published:** 2016-06-23

**Authors:** Weston A. Nichols, Brandon J. Henderson, Christopher B. Marotta, Caroline Y. Yu, Chris Richards, Dennis A. Dougherty, Henry A. Lester, Bruce N. Cohen

**Affiliations:** 1 Division of Biology & Biological Engineering, California Institute of Technology, Pasadena, California, United States of America; 2 Division of Chemistry & Chemical Engineering, California Institute of Technology, Pasadena, California, United States of America; 3 Department of Chemistry, University of Kentucky, Lexington, KY, United States of America; Weizmann Institute of Science, ISRAEL

## Abstract

A number of mutations in α4β2-containing (α4β2*) nicotinic acetylcholine (ACh) receptors (nAChRs) are linked to autosomal dominant nocturnal frontal lobe epilepsy (ADNFLE), including one in the β2 subunit called β2V287L. Two α4β2* subtypes with different subunit stoichiometries and ACh sensitivities co-exist in the brain, a high-sensitivity subtype with (α4)_2_(β2)_3_ subunit stoichiometry and a low-sensitivity subtype with (α4)_3_(β2)_2_ stoichiometry. The α5 nicotinic subunit also co-assembles with α4β2 to form a high-sensitivity α5α4β2 nAChR. Previous studies suggest that the β2V287L mutation suppresses low-sensitivity α4β2* nAChR expression in a knock-in mouse model and also that α5 co-expression improves the surface expression of ADNFLE mutant nAChRs in a cell line. To test these hypotheses further, we expressed mutant and wild-type (WT) nAChRs in oocytes and mammalian cell lines, and measured the effects of the β2V287L mutation on surface receptor expression and the ACh response using electrophysiology, a voltage-sensitive fluorescent dye, and superecliptic pHluorin (SEP). The β2V287L mutation reduced the EC_50_ values of high- and low-sensitivity α4β2 nAChRs expressed in *Xenopus* oocytes for ACh by a similar factor and suppressed low-sensitivity α4β2 expression. In contrast, it did not affect the EC_50_ of α5α4β2 nAChRs for ACh. Measurements of the ACh responses of WT and mutant nAChRs expressed in mammalian cell lines using a voltage-sensitive fluorescent dye and whole-cell patch-clamping confirm the oocyte data. They also show that, despite reducing the maximum response, β2V287L increased the α4β2 response to a sub-saturating ACh concentration (1 μM). Finally, imaging SEP-tagged α5, α4, β2, and β2V287L subunits showed that β2V287L reduced total α4β2 nAChR surface expression, increased the number of β2 subunits per α4β2 receptor, and increased surface α5α4β2 nAChR expression. Thus, the β2V287L mutation alters the subunit composition and sensitivity of α4β2 nAChRs, and increases α5α4β2 surface expression.

## Introduction

Autosomal dominant nocturnal frontal lobe epilepsy (ADNFLE) is a familial partial epilepsy linked to mutations in the α2, α4, and β2 nicotinic acetylcholine (ACh) receptor (nAChR) subunits [1, 2], and the Na^+^-gated K^+^ channel, *KCNT1* [3, 4]. Mutations in the corticotrophin releasing hormone [5], and the gene encoding the “Dishevilled, Egl-10, and Pleckstrin domain-coding protein 5” (*DEPDC5*) [6] are also associated with ADNFLE. ADNFLE patients suffer from brief nocturnal seizures that occur primarily during slow-wave (SW) sleep and originate in the frontal lobe. Seizures typically begin in childhood at 5–15 years and persist through adult life [7, 8]. The onset of ADNFLE seizures coincides with a shift in the location of maximal slow-wave activity (SWA) during sleep from the occipital to frontal lobe. This shift occurs around age 10 and persists throughout adult life [9]. Thus, the onset of ADNFLE seizures correlates closely with a sharp increase in the relative power of sleep-related SWA activity (1–4.5 Hz bandwidth) in the frontal lobe.

A number of ADNFLE mutations increase the sensitivity of α4β2 nAChRs to the endogenous agonist ACh and all mutations tested so far reduce allosteric Ca^2+^ potentiation of the α4β2 ACh response [1, 10]. Nicotinic receptors containing α4 and β2 subunits are distributed widely throughout the brain [11] but our understanding of their role in normal brain synaptic transmission and development is unfortunately limited. This limitation means that we cannot accurately predict which functional effects of the ADNFLE mutations are relevant to ictogenesis and other symptoms associated with the ADNFLE mutations. Thus, it is important to document all of their potentially relevant effects on nAChR function and expression.

β2V287L is a mutation in the second transmembrane domain (M2) of the β2 nAChR subunit linked to ADNFLE in a large Italian family [12, 13]. Affected family members exhibit brief nocturnal seizures (30–60 s) with hyperkinetic or tonic seizure semiology [13]. Ictal electroencephalographic (EEG) recordings reveal diffuse slow and sharp wave epileptiform activity [13]. Positron emission tomography further shows a reduction in nAChR density in the right dorsolateral prefrontal cortex of patients [14]. Previous studies of heterologously expressed α4β2 nAChRs show that the β2V287L mutation has a variety of effects on the functional properties of the receptors including changes in agonist sensitivity, agonist-induced desensitization, single-channel conductance, and allosteric Ca^2+^ potentiation [12, 15, 16].

Transgenic expression of the β2V287L mutation in rodents produces a variable seizure phenotype [17–20]. Variable phenotypes are expected given differences in genetic backgrounds of the animals used for these studies and the methods of transgene insertion, *i*.*e*., targeted [18, 19] and untargeted insertion [17, 20]. Targeted insertion preserves the location of the gene in the genome and its contiguity with adjacent regulatory domains. Untargeted insertions do not. Thus, transcriptional regulation of untargeted transgene insertions may be abnormal.

Mice with high copy numbers of untargeted β2V287L transgene insertions (β2V287L transgenic mice) display spontaneous epileptiform EEG activity during bouts of increased delta activity (presumably SW sleep) [17]. Interestingly, silencing the conditionally-expressed mutant transgene prevents epileptiform activity only if it is done during early development, suggesting a critical period for the development of β2V287L-induced seizures [17]. In contrast, mice with targeted β2V287L gene insertions (β2V287L knock-in mice) display increased mortality and behavioral abnormalities, but apparently not spontaneous epileptiform EEG activity [18, 19]. However, undetected epileptiform activity could occur in the β2V287L knock-in mice, particularly in light of their increased mortality. Seizures in these mice may be a rare event or occur primarily in older animals, which are not typically used for EEG recordings. Despite the apparent absence of spontaneous seizures, β2V287L knock-in mice exhibit a novel nicotine-induced seizure phenotype that involves tonic forelimb and digit extension [18], similar to that previously reported for α4S248F ADNFLE knock-in mice [21]. Nicotine-induced tonic forelimb and digit extension are rarely, if ever, observed in wild-type (WT) mice.

Measurements of total α4β2* nAChR protein in the β2V287L knock-in brains, and maximal ACh-induced ^86^Rb flux, [^3^H]dopamine release, and [^3^H]GABA release from mouse brain synaptosomes show that the β2V287L mutation reduces overall α4β2* nAChR expression [18]. Lower EC_50_ values for synaptosomal ACh-induced ^86^Rb flux, [^3^H]dopamine release, and [^3^H]GABA release show that it also increases α4β2* nAChR agonist sensitivity [18]. Thus, β2V287L reduces the total number of α4β2* nAChRs in knock-in mice but increases their ACh sensitivity.

Nicotinic α4 and β2 subunits co-assemble to form functional nAChRs with different α4:β2 subunit stoichiometries *in vitro* [22] and *in vivo* [23]. The two most common subunit stoichiometries are (α4)_2_(β2)_3_ and (α4)_3_(β2)_2_. The sensitivity of (α4)_2_(β2)_3_ receptors to ACh is ~100 times higher than that of (α4)_3_(β2)_2_ receptors [24]. The ACh concentration-response relation for α4β2*-mediated ^86^Rb efflux from cortical and thalamic synaptosomes is biphasic [23], suggesting the existence of both stoichiometries *in vivo*. Data from mice with partial α4 and β2 gene deletions further suggest that (α4)_2_(β2)_3_ receptors mediate high-sensitivity (HS) ACh-induced ^86^Rb syanptosomal release, and (α4)_3_(β2)_2_ receptors mediate low-sensitivity (LS) release [23].

ADNFLE mutations could increase the overall ACh sensitivity of the α4β2 nAChR population by two possible mechanisms. They could (1) increase the ACh sensitivity of individual receptors or (2) reduce the proportion of low-sensitivity (α4)_3_(β2)_2_ subtypes in the population. Here, we use a combination of fluorescent imaging and electrophysiological techniques to determine which of these mechanisms contributes to the effects of the β2V287L mutation on ACh sensitivity. The α4 and β2 nicotinic subunits also co-assemble with α5 in the brain to form α5α4β2 nAChRs [25]. Thus, we also asked whether β2V287L alters the expression or ACh sensitivity of α5α4β2 nAChRs. The results show that the β2V287L mutation increases overall α4β2 ACh sensitivity by both suppressing LS α4β2 expression, and increasing HS (and LS) α4β2 ACh sensitivity. In contrast, the β2V287L mutation increases α5α4β2 nAChR surface expression, but not ACh sensitivity. Thus, our data provide new information about how ADNFLE mutations alter the function and expression of brain nicotinic receptors.

## Materials and Methods

### Molecular biology

We used WT mouse α4, α5, and β2 cDNA clones inserted into the pCI-NEO vector to express α4β2 and α5α4β2 nAChRs in mammalian cell lines. To measure subunit protein expression in the plasma membrane (PM), C-terminal SEP tags were added to the α5 and β2 subunits using a previously described PCR protocol and primers that overlapped the C-terminals of the coding sequences [26]. The forward primer for the α5-SEP reaction was 5’-ACATTGGAAACACAATTAAGATGAGTAAAGGAGAAGAACT-3’, and the reverse primer was 5’-CGGGCCCTCTAGATCNTCAGGTTATTTGTATAGTTCATCCA -3’. The forward primer for the β2-SEP reaction was 5′-ACTCAGCTCCCAGCTCCAAGATGAGTAAAGGAGAAGAACT-3′ and the reverse primer was 5′- GGAGCTGCAAATGAGAGACCTTATTTGTATAGTTCATCCA-3′. After reaction completion, the resulting PCR products were cloned into a vector containing either the α5 or β2 cDNA clone using Pfu-Turbo polymerase. The mouse α4-SEP was constructed previously using the same protocol [26]. We used the QuikChange II XL site-directed mutagenesis kit with the forward primer 5′- CTGCTCATCTCCAAGATTCTGCCTCCCACCTCCCTCGACGTA-3′ and the reverse primer 5′-TACGTCGAGGGAGGTGGGAGGCAGAATCTTGGAGATGAGCAG-3′ to construct the β2V287L mutation. For expression in oocytes, mouse α5, α4 and β2 cDNA clones were inserted into the pGEMHE vector. The QuikChange protocol (Stratagene, La Jolla, CA) was used to construct site-directed mutations. To make mRNA for the oocyte injections, α4 and β2 cDNA plasmids were linearized using *SbfI*. The α5 plasmid was linearized using *SphI*. After DNA purification (Qiagen, Valencia, CA), we synthesized mRNA from the linearized DNA using the T7 mMessage Machine kit (Ambion, Grand Island, NY). The Qiagen RNeasy RNA purification kit was used to purify the synthesized mRNA.

### Tissue culture and transfection

Mouse Neuroblastoma-2a (N2a) cells were cultured using standard techniques and maintained in 50% Dulbecco’s Modified Eagle’s Medium (DMEM), 50% Opti-MEM and supplemented with 10% fetal bovine serum. N2a cells were plated by adding 90,000 cells to poly-d-lysine-coated 35-mm glass-bottom imaging dishes (MatTek Corp., Ashland, MA). One day after plating, plasmid DNA was mixed with cationic lipids by adding 500–750 ng of each nAChR plasmid to 4 μl of Lipofectamine 2000 transfection reagent in 250 μl of Opti-MEM. After 20 min at ambient temperature, the transfection mixture was added to N2a cells in 1 ml of Opti-MEM and incubated at 37°C for 24 h. Dishes were rinsed twice with N2a culture media and incubated at 37°C for another 24 h before imaging or performing electrophysiological experiments. Human embryonic kidney (HEK-293) cells were also cultured using standard cell techniques as above in DMEM supplemented with 10% fetal bovine serum. The cells were plated onto black-walled, clear-bottomed 96-well plates at a density of 50,000 cells per well. The following day, they were transfected with 100 ng of the appropriate cDNA mixtures combined with 4 uL of Lipofectamine 2000, as described above. Transfected cells were used 24 h later for the fluorescent membrane potential assay. The N2a and HEK-293 cell lines were purchased from the American Type Culture Collection (ATCC, Manassas, VA) in 2013 (cat. #’s CCL-131 & CRL-1573, respectively).

### Fluorescent membrane potential assay

We used a fluorescent plate reader (FlexStation III, Molecular Devices, Sunnyvale, CA) and a proprietary, membrane-potential-sensitive dye (Membrane Potential blue kit, Molecular Devices, Sunnyvale, CA) to measure the ACh concentration-response relations of nAChRs expressed in HEK cells. The cells were transfected with nAChR subunits and plated in V-shaped, 96-well plates. One day after plating, ACh-induced changes in membrane potential were visualized using the fluorescent plate reader and Membrane Potential Blue-Dye Kit (Molecular Devices). ACh was added to the wells at a speed of 16 μL/s. The peak response was measured in relative fluorescent units (RFU) and used to construct ACh concentration-response relations.

### Oocyte injections and electrophysiology

**Fig 1:**
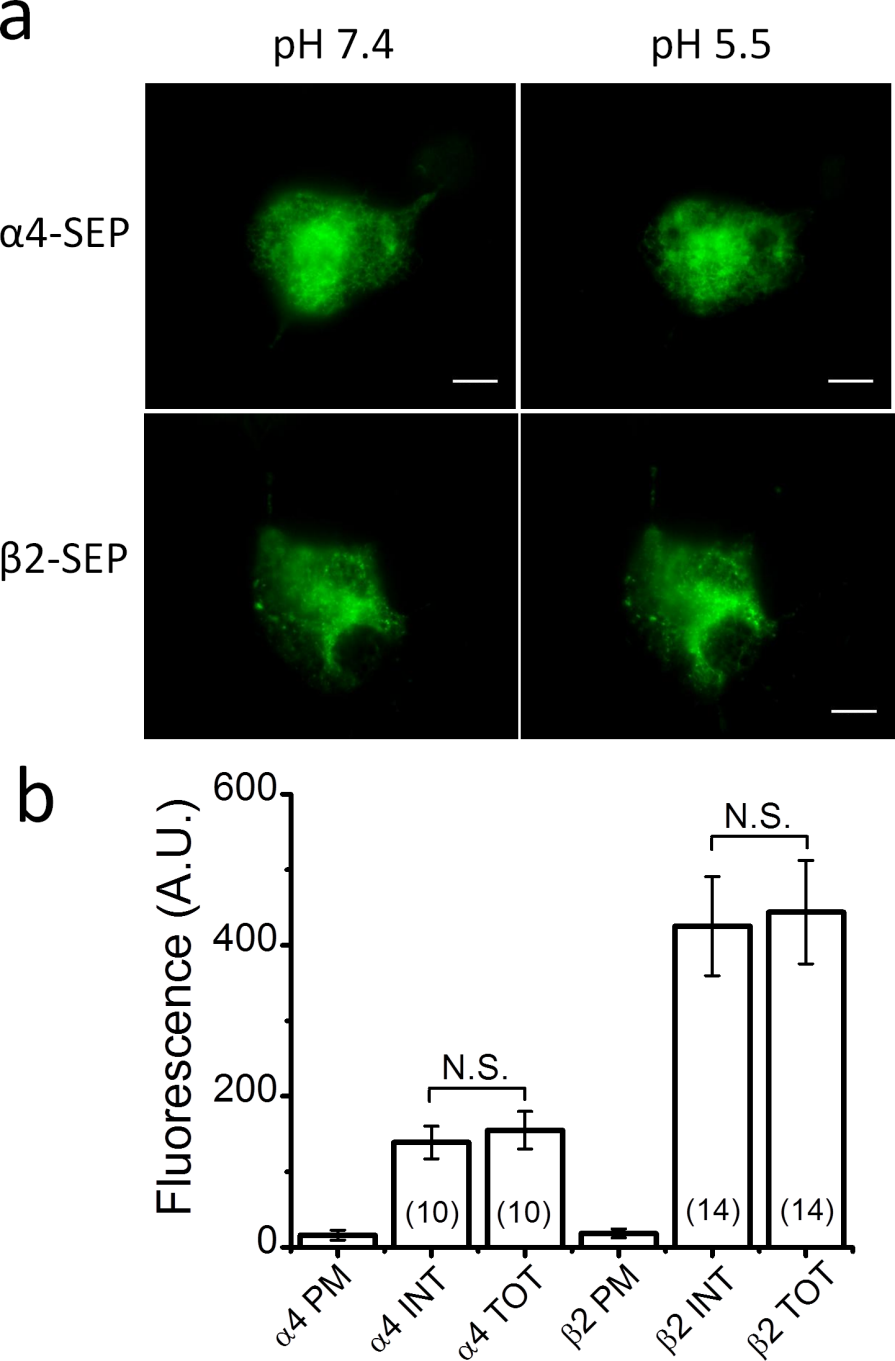
Transfection of N2a cells with just α4 or β2 fails to produce significant surface expression. Transfection of just α4 or β2 cDNA without the complementary subunit failed to produce significant α4 or β2 protein expression in the plasma membrane (PM). The α4 and β2 subunits were tagged with superecliptic pHluorin (SEP) and imaged with total internal reflection fluorescent (TIRF) microscopy. **a**. TIRF images of N2a cells expressing SEP-tagged α4 (α4-SEP) (top row) and β2 subunits (β2-SEP) (bottom row) at an extracellular pH of 7.4 (left column) and 5.5 (right column). The absence of a significant effect of extracellular pH on cellular fluorescence shows that SEP-tagged protein expression on the PM was negligible. Scale bars are 10 μm. **b**. The bars are the internal (INT), total (TOT), and PM fluorescent intensity measured in arbitrary units (A.U.) for N2a cells transfected with α4-SEP or β2-SEP cDNA. Internal and total fluorescence was not significantly different (N.S.) for the α4-SEP, or β2-SEP, subunit.

**Fig 2:**
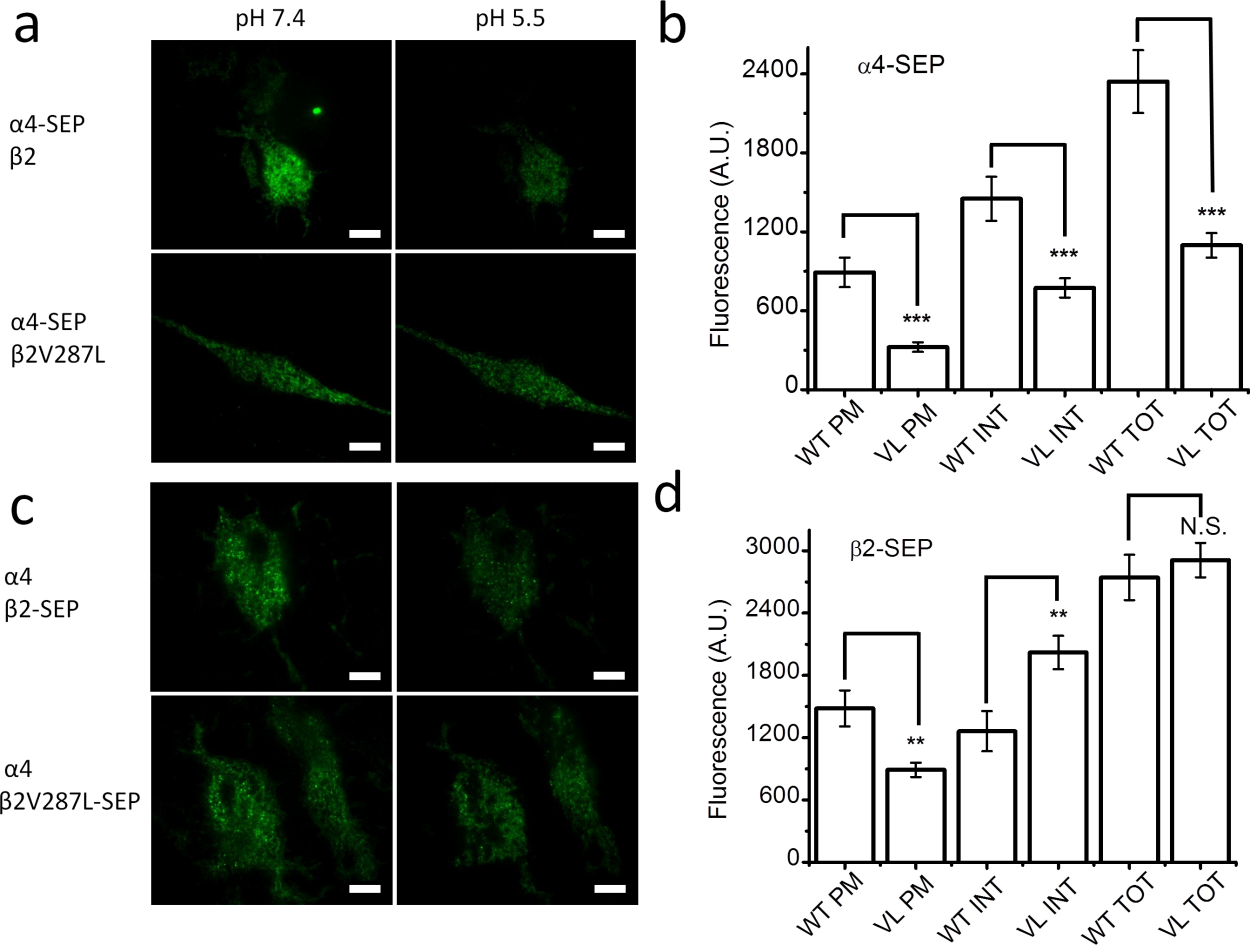
β2V287L alters the surface expression and subunit stoichiometry of SEP-labeled α4β2 nAChRs. **a.** TIRF images of α4-SEP fluorescence in N2a cells transfected with α4-SEPβ2 (top row), or α4-SEPβ2V287L, cDNA (bottom row) at an extracellular pH of 7.4 (left column) and pH 5.5 (right column). Reducing the pH from 7.4 to 5.5 dimmed cellular fluorescence because surface SEP-tagged subunits do not fluoresce at pH 5.5. Scale bars are 10 μm. **b.** The bars (from left to right) are the PM, INT, and TOT fluorescent intensities for cells transfected with α4-SEPβ2 (WT) or α4-SEPβ2V287L (VL) cDNA. The β2V287L mutation significantly (*p* < 0.001,***) reduced the TOT, INT, and PM α4-SEP fluorescence. **c.** Images of β2-SEP fluorescence in cells transfected with α4β2-SEP (top row), or α4β2V287L-SEP, cDNA (bottom row) at an extracellular pH of 7.4 (left column) and pH 5.5 (right column). **d**. The bars are the PM, INT, and TOT fluorescent intensities for cells transfected with α4β2-SEP (WT) or α4β2V287L-SEP (VL) cDNA. The β2V287L mutation significantly (*p* < 0.001) reduced PM β2-SEP fluorescence, but did not affect TOT fluorescence. It also significantly (*p* < 0.01,**) increased INT fluorescence.

This research was carried out in strict accordance with the recommendations in the Guide for the Care and Use of Laboratory Animals of the National Institutes of Health. We used a standard protocol approved by the Office of Laboratory Animal Research at the California Institute of Technology (1301-12G) to surgically harvest *Xenopus laevis* stage V and VI oocytes. Surgery was performed under ethyl 3-aminobenzoate methane sulfonate (MS222) anesthesia, and all efforts were made to minimize suffering. To express α4β2 nAChRs with varying proportions of HS and LS receptors, α4 and β2 mRNAs were mixed in a ratio of 1:1 or 10:1 to produce final concentrations of 0.13 ng/nL or 0.42 ng/nL, respectively. The oocytes were injected with a total of 6.7 ng or 21 ng of mRNA, respectively (in 50 nL). To express α4α5β2 nAChRs, we injected oocytes with a large excess of α5 mRNA. The α5, α4 and β2 subunits were mixed in a ratio of 10:1:1 (*w*/*w*/*w*) to produce a final concentration of 0.8 ng/nL. The oocytes were injected with 50 nL of this mixture (to deliver a total of 40 ng of mRNA per oocyte). After injection, the oocytes were incubated in ND96+ medium (96 mM NaCl, 2 mM KCl, 1.8 mM CaCl_2_, 1.8 mM MgCl_2_, 5 mM HEPES, 2.5 mM Na pyruvate, 0.6 mM theophylline, 50 μg/mL gentamycin, pH 7.4) at 18°C for 24–96 h. ACh chloride was purchased from Sigma-Aldrich (St. Louis, MO) and dissolved in a nominally Ca^2+^-free ND96 buffer (96 mM NaCl, 2 mM KCl, 1 mM MgCl_2_, 5 mM HEPES at pH 7.5). An automated two-electrode voltage-clamp (OpusXpress 6000A, Molecular Devices) was used to record ACh-induced currents from the injected oocytes at a holding potential of -60 mV. Ca^2+^-free ND96 was used as the recording buffer to avoid contamination of the ACh responses by Ca^2+^-activated Cl^-^ currents. The voltage-clamp currents were low-passed filtered at 5 Hz prior to digitization and sampled at 50 Hz. To measure the ACh response, 1 ml of the ACh-containing solution was applied to the oocyte for 15 s from a plastic pipette tip positioned next to it, followed by a 2 min buffer rinse. To obtain the ACh concentration-response relation, we applied 18 different ACh concentrations to each oocyte, recorded the responses, and normalized them to the maximum observed response for that oocyte. Because of the variability of oocyte expression, normalization of the data prior to pooling is a necessary, and standard, practice. Normalization has two advantages, (1) it reduces variance, and (2) it equalizes the weighting of data from different oocytes. Without normalization, data from oocytes with the highest expression levels would receive the heaviest weighting and bias the fits. To estimate the EC_50_ and maximum response, the pooled data were fit to a single rectangular hyperbola or the sum of two such hyperbolas using nonlinear least-squares regression. After fitting, the data in Figs 1E, 1F and 2C, were renormalized to the fitted maximum response to allow a direct visual comparison of the WT and mutant ACh sensitivities.

### Whole-cell patch clamping

Nicotinic receptors were expressed in N2a cells by co-transfecting the cells with an α4 nAChR subunit tagged with an enhanced green fluorescent protein (eGFP) insert (α4-eGFP) and, a WT or mutant β subunit. The fluorescent α4-eGFP subunit allowed us to target cells expressing nAChRs for recording [26]. We used an inverted fluorescence microscope (IX71, Olympus, Center Valley, PA) equipped with a high-pressure Hg lamp (HB-10103AF, Nikon, Melville, NY) to visualize the fluorescent cells. Whole-cell voltage-clamp currents were recorded using an Axopatch-1D amplifier (Molecular Devices) and digitized with the Digidata 1440A analog-to-digital converter (Molecular Devices), under the control of the pClamp 10.0 software (Molecular Devices). The pipette-filling solution contained (in mM): 135 K gluconate, 5 KCl, 5 EGTA, 0.5 CaCl_2_, 10 HEPES, 2 Mg-ATP, and 0.1 GTP (pH was adjusted to 7.2 with Tris-base, osmolarity to 280–300 mOsm with sucrose). Patch electrode input resistance was 3–5 MΩ. All recordings were obtained at ambient temperature. The data were low-passed filtered at 2 kHz and sampled at 10 kHz. ACh was dissolved in an extracellular solution containing (in mM): 140 NaCl, 5 KCl, 2 CaCl_2_, 1 MgCl_2_, 10 HEPES, and 10 glucose (320 mOsm, pH to 7.3 with Tris-base), and microperfused onto the cells using pressure ejection through a glass micropipette (300 ms pulse of 20 psi) (Picospritzer II, General Valve Corp., E. Hanover, NJ) or a commercially available microperfusion system (500 ms pulse of 6 psi) (Octaflow II, ALA Scientific Instruments, Farmingdale, NY). The holding potential was -50 mV. To minimize agonist-induced desensitization, we applied brief pulses of ACh (500 ms) at 3 min intervals, and continually perfused the recording chamber with saline.

### TIRF Microscopy

Live N2a cells were imaged 24 h after transfection in a stage-mounted culture dish incubator at 37°C (Warner Instruments, Hamden, CT). TIRF microscopy allowed us to visualize fluorescently-tagged proteins within 250 nm of the cell-coverslip interface. TIRF images were obtained using an inverted microscope (IX81; Olympus) equipped with an Olympus PlanApo 100× 1.45 NA oil objective and a digital stepper motor (Thorlabs, Newton, NJ) to translate a focused beam laterally across the back aperture of the objective for total internal reflection. The microscope included a drift control module that maintained samples at a constant focus for periods of ≥24 h. Neuronal medium was exchanged for a simpler extracellular solution (in mM: 150 NaCl, 4 KCl, 10 HEPES, 2 MgCl_2_, 2 CaCl_2_, and 10 glucose, pH 5.4 or 7.4) before imaging. SEP was excited using the 488-nm line of a multiline air-cooled argon laser (IMA101040ALS; Melles Griot, Carlsbad, CA). Images were captured with a back-illuminated EMCCD camera (iXON DU-897, Andor Technology USA, South Windsor, CT). Frame rates, laser power settings, and camera gain parameters were adjusted initially and maintained at a constant setting across all samples for each imaging session. Fluorescent intensity is reported in arbitrary units (A.U.). We acidified the imaging dish by perfusing the bath (normally pH 7.4) with an otherwise identical solution adjusted to pH 5.4. The ratio of PM to endoplasmic reticulum (ER) expression was determined by taking an initial TIRF image of each cell at pH 7.4 followed by acidification of the solution and a subsequent low-pH image. A region of interest encompassing just the cell was set to an internal threshold to specifically demarcate it. The ratio of the average intensities (fluorescence at pH 5.5/initial fluorescence at pH 7.4) indicates the fraction of PM fluorescence; smaller values imply higher PM expression.

### Statistical Analysis

Concentration-response relations for the ACh fluorescent, and voltage-clamp, responses were fit to the Hill equation using nonlinear least-squares regression (OriginLab software, Northampton, MA). Errors for the mean values reported in the text and figures are ± SEM. SEMs for the ratios of two mean values were calculated using a previously published approximation [27]. Statistical significance was determined by Student’s t test, or a one-way ANOVA followed by *post hoc* comparisons, when appropriate. Significant differences are reported at the level of *p*<0.05 (*), *p*<0.01 (**), or *p*<0.001 (***).

## Results

### β2V287L alters surface expression and stoichiometry

Previous data show that the β2V287L mutation suppresses the LS component of ACh-induced synaptosomal ^86^Rb^+^ release in a β2V287L knock-in mouse [18]. In principle, either a mutant-induced suppression of LS α4β2* nAChR surface expression, or an increase in LS ACh sensitivity, could account for this effect. To determine whether β2V287L affected the expression and subunit composition of surface α4β2 nAChRs, we constructed α4, β2, and β2V287L nAChR subunits with an extracellular SEP tag and measured the effects of the mutation on the expression of the SEP-tagged subunits in the ER and PM using TIRF microscopy. SEP is a pH-sensitive eGFP analog that does not fluoresce in acidic solutions. Thus, bathing cells in an acidic solution quenches the fluorescence of PM proteins with extracellular SEP tags, and the acid-induced reduction in total subunit fluorescence provides a way to estimate surface α4-, β2-, and β2V287L-SEP subunit expression [26, 28]. We used SEP-tagged nAChR subunits to determine whether the β2V287L mutation affected the number and subunit stoichiometry of α4β2 receptors expressed in the PM.

To ensure that the α4 and β2 subunits expressed in the PM were indeed incorporated into α4β2 receptors, we first asked whether individual α4 and β2 SEP-tagged subunits are expressed on the cell surface without co-transfection of the complementary subunit (Fig 1). The answer was an unambiguous no. Transfection with either WT α4-SEP or β2-SEP alone resulted in readily detectable fluorescent protein inside the cell but practically none on the PM (Fig 1B). Total and internal fluorescence did not differ significantly for either α4-SEP or β2-SEP (Fig 1B). Thus, without the complementary subunit, virtually all the α4 and β2 protein remains in the intracellular ER.

We then measured the effects of the β2V287L mutation on α4-SEP and β2-SEP expression in cells transfected with both α4 and β2 subunits (Fig 2). The β2V287L mutation (labeled simply as “VL” in Fig 2) significantly (*p* < 0.001) reduced total, ER, and PM α4-SEP cellular fluorescence (Fig 2B). In contrast, it did not significantly affect total β2-SEP fluorescence, but it did significantly (*p* < 0.01) increase ER, and reduce PM, β2-SEP fluorescence (Fig 2D). Summarizing, β2V287L significantly reduced PM expression of both the α4 and β2 subunits (Fig 2B and 2D). However, surface fluorescence for the α4-SEP subunit of the mutant receptors was 36 ± 6% (n = 54 cells) that of the WT receptors, whereas surface fluorescence of the β2V287L-SEP subunit was 60 ± 8% (n = 54) that of the WT β2-SEP subunit. Thus, even though the mutation reduced the surface expression of both subunits, the fractional reduction in α4 was greater than that for β2. If the mutation simply reduced the number of surface receptors without altering their subunit stoichiometry, then the number of α4-SEP and β2V287L-SEP subunits in the mutant receptors would decline by the same percentage. However, because there was a significantly greater (*p* < 0.05) decrease in the percentage of α4 on the cell surface than β2, the β2V287L mutation must have reduced both the number of α4 subunits incorporated into individual surface nAChRs (*i*.*e*., altered subunit stoichiometry), and the total number of surface α4β2 nAChRs. The greater reduction in α4 subunits suggests that mutation suppresses surface expression of the (α4)_3_(β2)_2_ stoichiometry, which is the subunit stoichiometry of LS α4β2 nAChRs.

We can estimate the mutant-induced reduction in the total surface receptor expression, and the proportion of surface HS receptors in the WT population, from these data by making two simplifying assumptions. First, we assume that the α4:β2 subunit ratio for the WT HS receptors is 2:3, whereas that for the LS receptors is 3:2. This assumption is consistent with previous data [22, 24]. Second, we assume that mammalian cell lines transfected with equal amounts of α4 and β2V287L cDNA (w/w) express only HS receptors in the plasma membrane, and their α4:β2 subunit ratio is 2:3 (identical to WT HS receptors). Using these two assumptions, the number of surface WT α4 subunits (*α*_*WT*_) is then:

$$\alpha_{WT}=T_{WT}[2(x)+3(1-x)],$$

where *T*_*WT*_ is the total number of α4β2 surface receptors, *x* is the fraction of HS receptors, 2(*x*) is the number of α subunits in surface HS receptors, and 3(1-*x*) is the number in surface LS receptors. The number of mutant surface α4 subunits (*α*_*M*_) is simply:

$$\alpha_{M}={2T}_{M},$$

where *T*_*M*_ is the total number of mutant α4β2V287L surface receptors. Taking the ratio of these two values (*α*_*M*_/*α*_*WT*_) and simplifying, we have:

$$\frac{\alpha_{M}}{\alpha_{WT}}=\frac{{2T}_{M}}{T_{WT}(3-x)}.$$ Eq 1

The number of surface WT β2 subunits (*β*_*WT*_) is:

$$\beta_{WT}=T_{WT}[3(x)+2(1-x)].$$

The number of mutant surface β2V287L subunits (*β*_*M*_) is:

$$\beta_{M}={3T}_{M}.$$

Taking the ratio of *β*_*M*_ to *β*_*WT*_ and simplifying, we have:

$$\frac{\beta_{M}}{\beta_{WT}}=\frac{{3T}_{M}}{T_{WT}(2+x)}.$$ Eq 2

Dividing *α*_*M*_/*α*_*WT*_ by *β*_*M*_/*β*_*WT*_, we obtain:

$$\frac{\frac{\alpha_{M}}{\alpha_{WT}}}{\frac{\beta_{M}}{\beta_{WT}}}=\frac{2(x+2)}{3(x-3)}$$ Eq 3

Solving Eq 3 for *x* yields:

$$x=\frac{3\frac{\alpha_{M}\beta_{WT}}{\alpha_{WT}\beta_{M}}-\frac{4}{3}}{\frac{\alpha_{M}\beta_{WT}}{\alpha_{WT}\beta_{M}}+\frac{2}{3}}$$ Eq 4

Substituting the mean values for *α*_*M*_/*α*_*WT*_ (0.36) and *β*_*M*_/*β*_*WT*_ (0.6) obtained above in Eq 4 gives a value of 0.37 for *x*. Solving Eq 2 for *T*_*M*_*/T*_*WT*_, we have:

$$\frac{\left(x+2\right)}{3}\frac{\beta_{M}}{\beta_{WT}}=\frac{T_{M}}{T_{WT}}.$$ Eq 5

Substituting a value of 0.37 for *x* and 0.6 for *β*_*M*_/*β*_*WT*_ in Eq 2 gives a value of 0.47 for *T*_*M*_*/T*_*WT*_. According to this analysis, the β2V287L mutation reduced the total number of α4β2 nAChRs expressed on the plasma membrane of HEK cells by 53% ((1–0.47) X 100) and HS receptors account for 37% of the WT α4β2 nAChRs expressed in the plasma membrane of HEK cells.

### Mutation suppresses (α4)_3_(β2)_2_ functional expression

To confirm the results of the fluorescent imaging experiments (above), we expressed mouse α4β2 and α4β2V287L nAChRs in *Xenopus* oocytes using α:β mRNA injection ratios of 1:1 and 10:1, and measured the ACh concentration-response relations of the expressed receptors using an automated voltage-clamp. We used oocytes for these experiments because mRNA can be directly injected into the ooplasm. Thus, the subunit stoichiometry of the expressed receptors could be manipulated by simply biasing the α:β mRNA injection ratio. Previous studies show that injecting oocytes with equal amounts of WT α4 and β2 mRNAs produces a mixture of LS and HS α4β2 nAChRs. In contrast, injecting them with an excess of α4 mRNA favors the formation of LS receptors because they contain more α4, than β2, subunits [22, 24].

**Fig 3:**
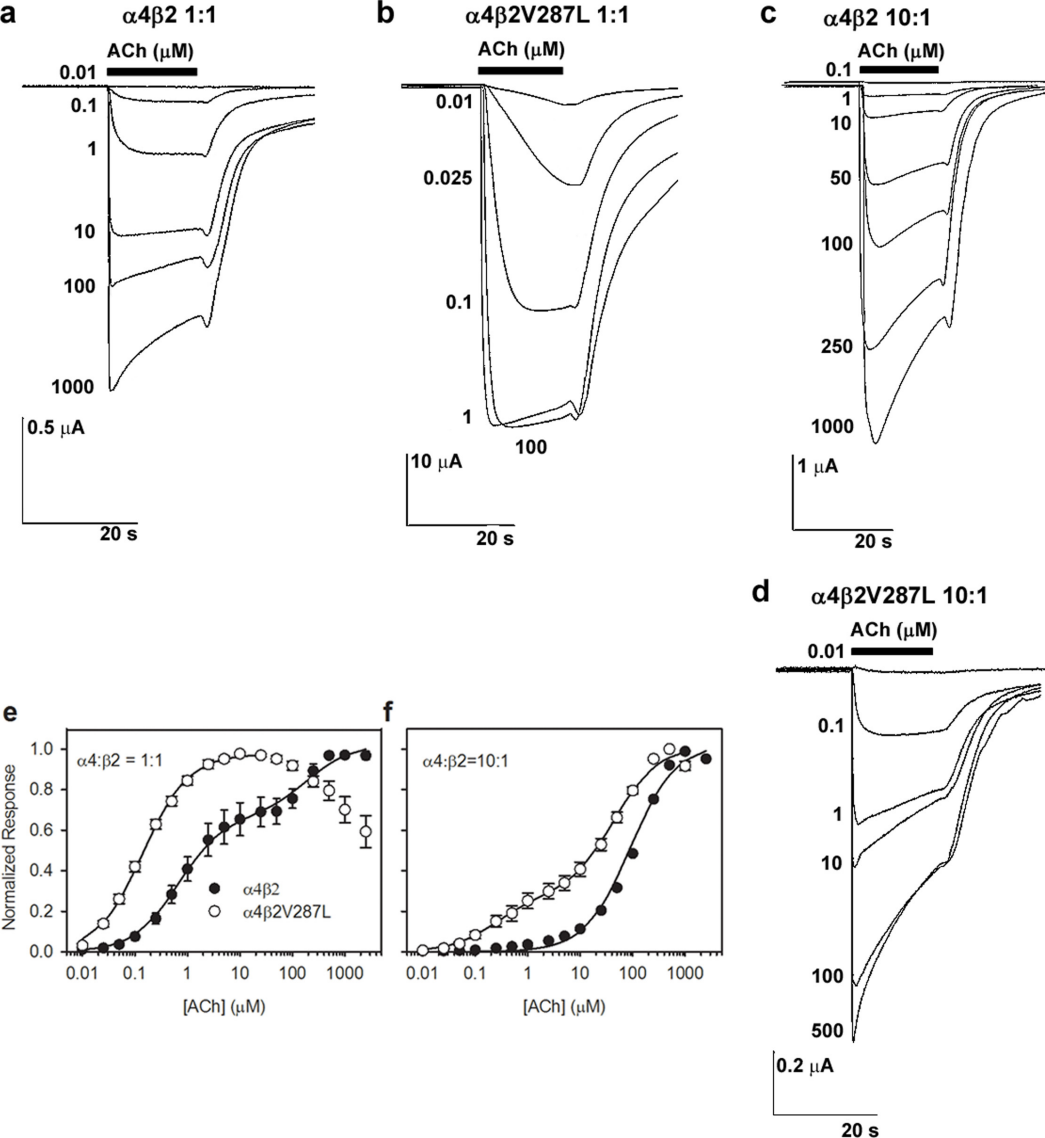
WT and mutant ACh concentration-response relations using unbiased and α4-biased subunit injection ratios. The α4β2 and α4β2V287L receptors were expressed in *Xenopus* oocytes using either a 1:1 (**a-b, e**) or 10:1 α4:β2 mRNA injection ratio (**c-d, f**). **a-b**. the traces are voltage-clamped ACh responses of oocytes injected with α4β2 (**a**) or α4β2V287L (**b**) in a 1:1 α4:β2 stoichiometric ratio (*w/w*). The ACh concentrations (in μM) are listed on the left of, or below, the traces. The bars above the traces show the timing and duration of the ACh application. The downward deflections of the trace are ACh-induced inward currents. For clarity, only a subset of the 18 responses recorded from each oocyte is shown. **c-d**, Voltage-clamped α4β2 (**c**) and α4β2V287L responses (**d**) using a 10:1 α4:β2 injection ratio. **e-f**. Normalized ACh concentration-relations for the α4β2 (filled circles) and α4β2V287L nAChRs (open circles) using 1:1 (**e**) and 10:1 (**f**) α4:β2 injection ratios. Lines are fits to the sum of two hyperbolic binding components using non-linear least-squares regression, subject to the constraints described in the text (Results). The data or individual oocytes were normalized to the observed maximum response for each oocyte, pooled across oocytes, and re-normalized to the fitted maximum response (Methods). Symbols are the means of 7–17 oocytes. Error bars are SEMs in this, and subsequent, figures (obscured by the symbols at some ACh concentrations). Holding potential = -60 mV.

Consistent with previous studies, we found that injecting oocytes with a 1:1 ratio of α4:β2 WT mRNA produced a biphasic concentration-response relation over a range of 0.01–2,500 μM ACh (Fig 3A and 3E). The biphasic concentration-response relation was fit to the sum of two hyperbolic binding components,

$$NR=\frac{A_{HS}}{1+\frac{{EC}_{50(HS)}}{\left[ACh\right]}}+\frac{A_{LS}}{1+\frac{{EC}_{50(LS)}}{\left[ACh\right]}},$$ Eq 6

where *NR* is the mean normalized peak response (see Methods), *A*_*HS*_ and *A*_*LS*_ are the amplitudes of the high- and low-sensitivity components, *EC*_*50(HS)*_ and *EC*_*50(LS)*_ are the EC_50_ values for the high- and low-sensitivity components, and *[ACh]* is the ACh concentration. Fitting the data to this equation gave EC_50_ values for the two components that differed >tenfold (Table 1). The EC_50_ value of the HS component was 0.67 ± 0.08 μM and that of the LS component was 190 ± 60 μM (n = 18 ACh concentrations). The HS component accounted for 65 ± 2% (n = 18 concentrations) of the maximum WT response (Table 1). In contrast to the WT, injecting oocytes with a 1:1 ratio of α4 WT and β2V287L mutant mRNA produced a bell-shaped ACh concentration-response relation over this concentration range that clearly lacked an LS component (Fig 3B and 3E). Nicotinic bell-shaped concentration-response relations are typically attributed to agonist-induced desensitization or channel-block at the upper end of the agonist concentration range. However, the α4β2V287L traces in Fig 3B for the 1:1 α4:β2 mRNA injection ratio display little desensitization during the 15 s agonist applications, even at ACh concentrations greater than 100 μM. Thus, channel-block by ACh itself[29], rather than desensitization, most likely accounts for the reduction in peak response observed at these higher ACh concentrations.

**Table 1:** Fitted parameters for α4β2, α4β2V287L, α5α4β2, and α5α4β2V287L ACh concentration-response relations in oocytes.

Receptor	α:β mRNA Ratio	Component	% of Total	EC_50_ (μM)
α4β2	1:1	HS^a^	65 ± 2 (8)^b^	0.67 ± 0.08 (8)
		LS	35 ± 2 (8)	190 ± 60 (8)
α4β2V287L	1:1	HS	100^c^	0.11 ± 0.01 (8)***
α4β2	10:1	HS	7 ± 4 (17)	0.67 (fixed)^d^
		LS	93 ± 4 (17)	190 (fixed)
α4β2V287L	10:1	HS	23 ± 3 (7)**^e^	0.11 (fixed)
		LS	77 ± 3 (7)	36 ± 7 (7)*
α5α4β2	10:1:1	HS	100	0.26 ± 0.04 (4)
α5α4β2V287L	10:1:1	HS	100	0.32 ± 0.01 (8)

^a^HS and LS denote the high- and low-sensitivity components of the concentration-response relations, respectively.

^b^Values are mean ± S.E. (number of oocytes).

^c^For single-component concentration-response relations the percent of total (% of total) was fixed at 100%.

^d^Parameters constrained to a particular value are denoted as (fixed).

^e^Mutant values that differ significantly from WT (α4β2) at the 0.05. 0.01, and 0.001 levels are marked (*), (**), and (***).

The rising phase of the mutant concentration-response relation between 0.01 and 20 μM ACh was adequately fit by a single hyperbolic component with an EC_50_ value of 0.11 ± 01 μM (n = 11 concentrations) (Fig 3B and 3E). The EC_50_ value was significantly (*p* < 0.001) less than that of the WT HS component by a factor of six-fold (Table 1). Thus, for the 1:1 α4:β2 mRNA injection ratio, the β2V287L mutation shifted the ACh concentration-response relation leftward and eliminated the LS component.

Two mechanisms could potentially account for the effects of the β2V287L mutation on the 1:1 α:β ACh concentration-response relation. The mutation could (1) suppress LS receptor expression or (2) raise the ACh sensitivity of LS receptors to the point where they are indistinguishable from HS receptors. To test these hypotheses, we injected oocytes with an α:β mRNA ratio of 10:1 (which favors LS receptor expression) and measured the resulting WT and mutant concentration-response relations. The results show that α4 and β2V287L subunits can form functional LS nAChRs (Fig 3D and 3F). The absence of a detectable LS component from the 1:1 α:β mutant concentration-response relation strongly suggests that the mutation suppresses the expression of LS α4β2V287L nAChRs (Table 1).

Consistent with previous studies [22, 24], injecting WT α4:β2 mRNAs in a 10:1 ratio shifted the ACh concentration-response relation rightward (Fig 3C and 3F). The WT 10:1 ACh concentration-response relation was practically monophasic with a possible small HS component (Fig 3F). In contrast, the mutant 10:1 ACh concentration-response relation was clearly biphasic (Fig 3D and 3F). To statistically compare the relative amplitudes of the WT and mutant HS components, we fit both data sets to the sum of two hyperbolic components and reduced the number of free parameters by constraining the EC_50_ values of the two components to those obtained from fitting the 1:1 α4:β2 data (Fig 3F, Table 1). Previous results show that, at least for WT α4β2 nAChRs, unconstrained fits produce nearly equivalent EC_50_ values for the monophasic concentration-response relation with an excess of β2 (1:4 α4:β2), and for the HS component of the biphasic concentration-response relation with an excess of α4 [30]. Thus, as a curve-fitting assumption, this constraint appears reasonable.

Using this constraint, the percentage amplitude of the WT LS component was 93 ± 4% and that of the HS component was 7 ± 4% (n = 18 concentrations, Table 1). Although we could have fit the WT data to a single hyperbolic binding function (in which case the relative amplitude of the HS component would be zero), previous data show that injecting oocytes with WT α4:β2 mRNAs in a 4:1 ratio produces a biphasic ACh concentration-response relation with a small (16%) residual HS component[30]. This component appears to represent the activation of (α4)_3_(β2)_2_ receptors by two agonist molecules, rather than residual (α4)_2_(β2)_3_ receptor expression [30]. Regardless of the interpretation of the data in this particular case, our analysis puts an upper limit of 7 ± 4% on the possible percentage of residual (α4)_2_(β2)_3_ receptors in the WT population using a 10:1 α4:β2 mRNA injection ratio.

As in the WT analysis, we constrained the EC_50_ value for the mutant 10:1 α4:β2 HS component to the value obtained previously from the 1:1 α4:β2V287L fit (Table 1). The EC_50_ value for the LS component was free to vary. Using this constraint, the HS component accounted for 23 ± 3% of the maximum response, and the LS, for 77 ± 3% (n = 16 concentrations, Table 1). The mutant 10:1 concentration-response relation was well fit using this constraint (Fig 3F), suggesting that (similar to the WT) the EC_50_ value for the monophasic concentration-response relation with an excess of β2 was nearly the same as that for the HS component of the biphasic concentration-response relation with an excess of α4. The mutation significantly (p < 0.01) increased the percentage amplitude of the HS component compared to the WT 10:1 data (Table 1). It also significantly (*p* < 0.05) reduced the EC_50_ value for the LS component (36 ± 7 μM, n = 11 concentrations) by a factor of five-fold relative to the WT (Table 1).

There are two possible interpretations for the increased amplitude of the mutant HS component in the 10:1 α:β concentration-response relation. The mutation could increase (1) residual expression of receptors with the (α4)_2_(β2)_3_ stoichiometry or (2) the proportion of receptors with the (α4)_3_(β2)_2_ stoichiometry that are activated by binding two agonist molecules. The available data are not sufficient to exclude either interpretation. Nevertheless, the presence of a LS component in the mutant 10:1 α:β concentration-response relation, and its absence from the 1:1 α:β concentration-response relation, clearly suggest that the β2V287L mutation suppresses the expression of receptors with the (α4)_3_(β2)_2_ stoichiometry. Thus, consistent with the SEP data (above), the oocyte data show that β2V287L inhibits functional LS expression in oocytes. They also show that the mutation increases the ACh sensitivities of both the HS and LS subtypes by a similar factor.

### β2V287L does not affect α5α4β2 ACh sensitivity

**Fig 4:**
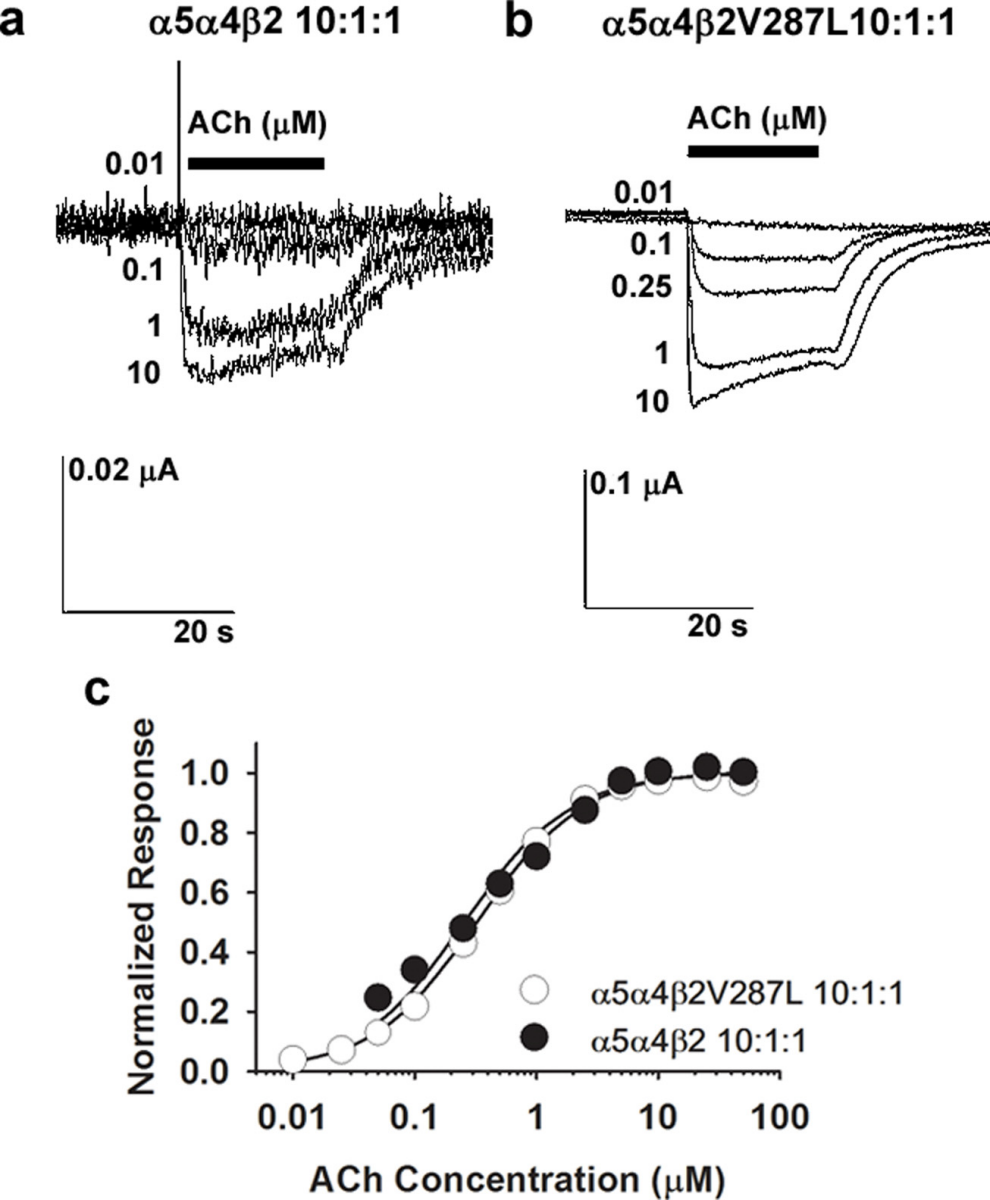
ACh concentration-response relations for α5α4β2 and α5α4β2V287L nAChRs. The α5α4β2 and α5α4β2V287L receptors were expressed in *Xenopus* oocytes using a large excess of α5 mRNA (α5:α4:β2 mRNA injection ratio of 10:1:1 *w/w/w*) to ensure that α5-containing (α5*) nAChRs were the predominantly expressed subtype. **a-b.** Voltage-clamped ACh responses of oocytes expressing α5α4β2 (**a**) or α5α4β2V287L receptors (**b**). **c**. Normalized ACh concentration-relations for the WT α5α4β2 (filled circles), and mutant α5α4β2V287L (open circles), nAChRs superimpose. The lines are fits to a single hyperbolic binding component using non-linear least-squares regression. The data for both receptors were normalized to the fitted maximum response to facilitate comparison of their ACh sensitivities. Symbols are the means of 4–8 oocytes. Holding potential = -60 mV.

The cortex and other CNS regions contain α5* nAChRs [31]. To determine whether the β2V287L mutation affected the ACh sensitivity of α5α4β2 nAChRs, we evaluated the ACh concentration-response relations of oocytes injected with either α5:α4:β2 (WT) or α5:α4:β2V287L (mutant) mRNA. The oocytes were injected with a large excess of α5 (α5:α4:β2 ratio of 10:1:1) to favor α5α4β2 co-assembly (Fig 4). The α5α4β2 WT and mutant ACh concentration-response relations were both monophasic, and adequately fit by a single hyperbolic component (Fig 4C). The EC_50_ values for the WT and mutant were 0.26 ± 0.04 μM (n = 10 concentrations) and 0.32 ± 0.01 μM (n = 12), respectively, and were within the range of EC_50_ values for the mutant and WT α4β*2* HS component (0.11–0.67 μM, Table 1). Thus, the β2V287L mutation did not affect the EC_50_ value for α5α4β2 nAChRs, even though it significantly reduced the EC_50_ values for the HS and LS α4β2 nAChRs (Table 1). The α5 subunit can occupy the accessory subunit position in the α5α4β2 receptor or replace a β2 subunit at one, of the two, canonical agonist binding sites [32, 33]. Thus, the replacement of β2V287L by α5 at one (or both) of these subunit positions prevents the mutation from affecting the ACh sensitivity of the assembled receptor.

### Mutation reduces functional α4β2 expression in HEK cells

**Fig 5:**
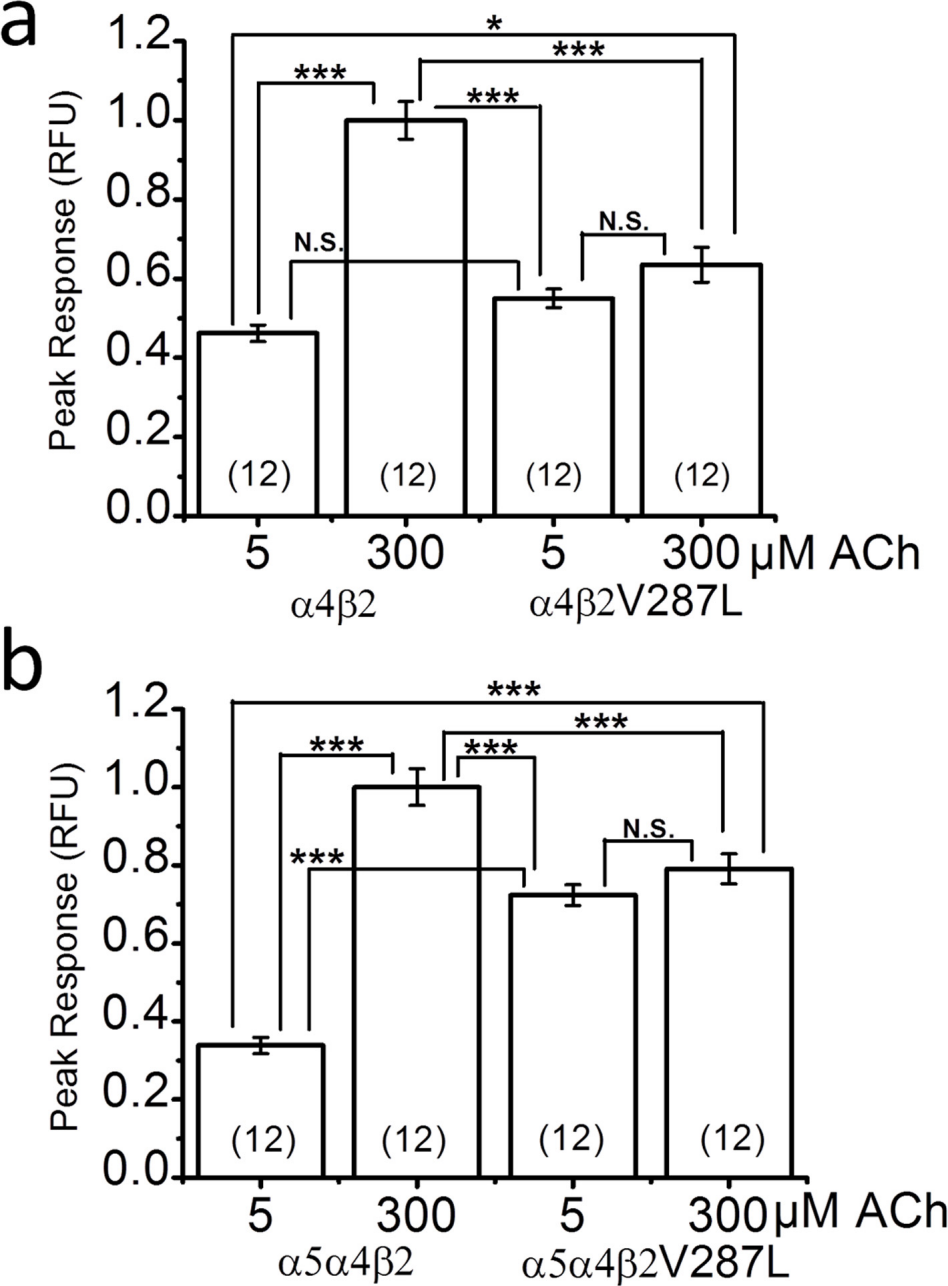
Fluorescent responses of α4β2, α4β2V287L, α5α4β2, and α5α4β2V287L nAChRs to 5 and 300 μM ACh. ACh responses were measured using a membrane-potential-sensitive fluorescent dye. **a**. The bars are peak responses to 5 and 300 μM ACh of α4β2 (WT) and α4β2V287L (mutant) receptors (measured in relative fluorescent units (RFU)). HEK cells were transfected with α4 and β2 cDNA in a 1:1 stoichiometric ratio (*w/w*). The WT and mutant responses were measured in matched groups of cells transfected on the same day, incubated for the same time, and tested on the same day. All responses were normalized to the peak WT 300 μM ACh response. Sample sizes (n = number of culture wells) are given in parentheses inside the bars. Connecting lines above the bars in this, and subsequent, figures indicate statistical comparisons between various groups. Asterisks give the significance levels for *post hoc* comparisons between the groups. Not significant (N.S.) **b**. Similar analysis for HEK cells transfected with α5α4β2 (WT), or α5α4β2V287L (mutant), cDNA in a 1:1:1 α5:α4:β2 stoichiometric ratio.

Previous results show that the β2V287L mutation reduces the maximum ACh response of synaptosomal α4β2* nAChRs from a β2V287L knock-in mouse [18]. To determine whether the mutation had a comparable effect on expressed α4β2 nAChRs, we transfected HEK cells with α4β2 or α4β2V287L cDNAs, and measured the responses of the expressed receptors to 5 and 300 μM ACh using a fluorescent membrane-potential-sensitive dye (Fig 5A). All responses were normalized to the WT 300 μM ACh response (Fig 5A). We chose these two ACh concentrations because 5 μM was close to the overall EC_50_ value for the WT 1:1 α:β ACh concentration-response relation measured in oocytes, and 300 μM was close to saturation (Fig 3E). In contrast, both 5 and 300 μM ACh were well above the EC_50_ value for mutant 1:1 α:β ACh concentration-response relation in oocytes (Fig 3F). Consistent with the oocyte data, the peak 5 μM ACh response of WT α4β2 nAChRs expressed in HEK cells was roughly half that of the 300 μM response (Fig 5A). In contrast, the peak 5 μM ACh response of the α4β2V287L nAChRs did not differ significantly from the peak 300 μM response, showing that 5 μM gave nearly a maximum response (Fig 5A). The mutant 300 μM ACh response was roughly 40% smaller than that of the WT (Fig 5A). Thus, β2V287L reduced the near-maximal ACh response of α4β2 nAChRs expressed in a mammalian cell line and increased the ACh sensitivity of the response. The similarity of the normalized 5 μM α4β2, 5 μM α4β2V287L, and 300 μM α4β2V287L responses is consistent with mutant suppression of the (α4)_3_(β2)_2_ stoichiometry (Fig 5A).

### α5 increases functional mutant expression in HEK cells

Previous data show that co-transfecting HEK cells with the ADNFLE mutant subunit α4S247F and the WT β2 subunit does not produce functional nAChRs unless an accessory nicotinic subunit such as α5 is added to the mix [34]. Our data show that HEK cells express functional α4β2V287L nAChRs without any additional subunits (above), but the 300 μM ACh α4β2V287L response is considerably less than that of the WT (Fig 5A). To determine whether adding α5 improves functional α4β2V287L expression, we transfected HEK cells with α5α4β2 or α5α4β2V287L cDNA in a 1:1:1 α5:α4:β2 ratio, and measured the response of the transfected cells to 5 and 300 μM ACh using a fluorescent membrane-potential-sensitive dye. The responses were normalized to the 300 μM ACh α5α4β2 response (Fig 5B). The normalized 5 μM ACh response of the α5α4β2-transfected cells (Fig 5B) was actually less than that of the α4β2-transfected cells (Fig 5A). Thus, most of the nAChRs expressed in the α5α4β2-transfected cells still appeared to be α4β2. Otherwise, because of the low EC_50_ value for α5α4β2 nAChRs (0.26 ± 0.04 μM, Table 1), we would expect the normalized 5 μM response of the α5α4β2-transfected cells to be larger than that of the α4β2-transfected cells. Similar to the mutant transfections without α5 (Fig 5A), the normalized 5 μM and 300 μM ACh responses of the α5α4β2V287L-transfected cells were not significantly different (Fig 3B). However, the addition of α5 significantly (*p* < 0.05) increased both the normalized 5 μM (*p* < 0.001) and 300 μM mutant responses (*p* < 0.05), compared to their respective WT values (Fig 5). For example, the normalized 300 μM ACh response of α5α4β2V287L-transfected cells was 0.79 ± 0.05 (n = 24 replicates), compared to 0.64 ± 0.05 (n = 24) for α4β2V287L-transfected cells. Thus, α5 inclusion significantly improved α4β2V287L functional expression in HEK cells.

### Mutation reduces EC_50_ and maximum response in HEK cells

**Fig 6:**
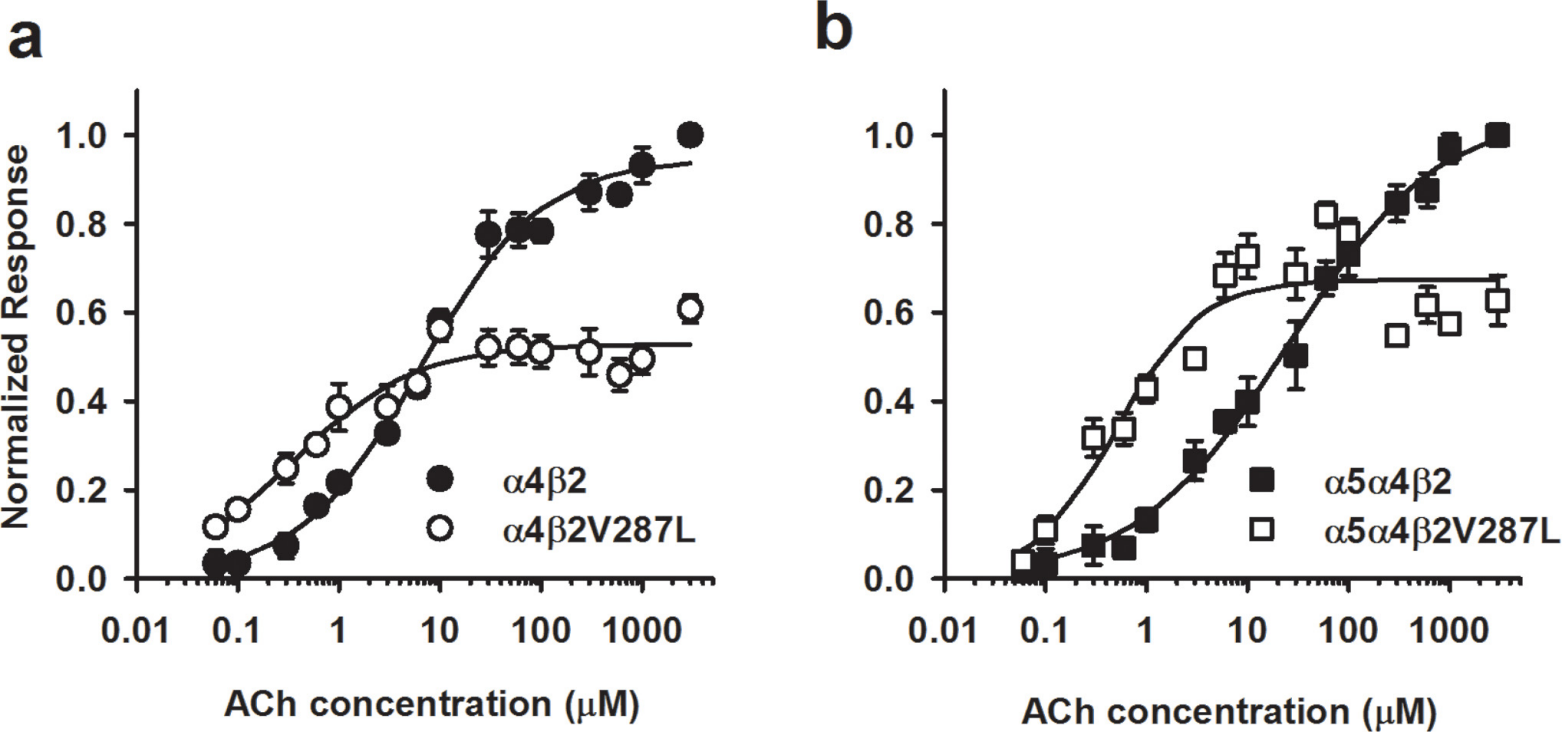
ACh concentration-response relations for α4β2, α4β2V287L, α5α4β2, and α5α4β2V287L nAChRs using a membrane-potential dye. **a.** β2V287L shifted the α4β2 ACh concentration-response to the left and reduced the maximum response. The data were normalized to the 1 mM ACh α4β2 response. The filled circles are α4β2 responses, and the open circles, α4β2V287L responses. The lines are fits to the three-parameter Hill equation (see Table 2 for the fitted parameters). **b**. Co-expression with α5 increased the maximum response of cells transfected with the mutant receptor cDNA (α5α4β2V287L) relative to the WT control (α5α4β2). The data were normalized to the 1 mM ACh α5α4β2 response. The α5:α4:β2 cDNA transfection ratio was 1:1:1 (*w/w*). The filled squares are α5α4β2 responses, and the open squares, α5α4β2V287L responses. All else was the same as in **a**.

Finally, we measured complete ACh concentration-response relations for HEK cells transfected with α4β2 (1:1 ratio), α4β2V287L (1:1), α5α4β2 (1:1:1), and α5α4β2V287L (1:1:1) cDNAs using the fluorescent membrane-potential-sensitive dye (Fig 6). The α4β2 concentration-response relation measured with the fluorescent dye (Fig 6A) had a shallow slope and was not as clearly biphasic as the corresponding voltage-clamp data in oocytes (Fig 3E). This difference may be because, in contrast to voltage-clamp responses, ACh-induced voltage responses are not linearly proportional to the number of open nAChRs throughout the effective ACh concentration range. Voltage responses become progressively smaller as the membrane potential approaches the nAChR reversal potential. Because of this distortion, instead of fitting the fluorescent-dye concentration-response relation to the sum of two hyperbolic components (Eq 6), we fit it to a three-parameter Hill equation (Eq 7),

$$NR=\frac{R_{max}}{1+{\left(\frac{{EC}_{50}}{\left[ACh\right]}\right)}^{n}},$$ Eq 7

where *NR* in this case is the mean fluorescent peak response normalized to the mean α4β2 value at 1 mM ACh, *R*_*max*_ is the maximum normalized response, *[ACh]* is the ACh concentration, *EC*_*50*_ is the ACh concentration at the half-maximal response, and *n* is the Hill coefficient. The fluorescent-dye data confirm that the β2V287L mutation reduces the EC_50_ and maximum response of α4β2 nAChRs expressed in a mammalian cell line (Table 2). Interestingly, α5 co-transfection significantly increased the WT EC_50_ in these experiments, presumably by adding a high-sensitivity α5α4β2 population to the existing α4β2 receptor pool (Table 2). Co-transfection with α5 also increased the relative amplitude of the mutant response compared to the corresponding WT transfections (α4β2, α5α4β2) at ACh concentrations in the 5–10 μM range, and ≥ 300 μM (Fig 6); however, it did not affect the mutant EC_50_ value (Table 2). The Hill coefficients of the α4β2, α4β2V287L, α5α4β2, and α5α4β2V287L concentration-response relations were not significantly different (Table 2). All of them (except α5α4β2V287L) were less than unity, indicating receptor heterogeneity (Table 2). For the α4β2 and α4β2V287L receptors at least, these low Hill coefficients suggest that the cells express a mixed population of receptors, containing both (α4)_2_(β2)_3_ and (α4)_2_(β2)_3_ subunit stoichiometries. The leftward shift and reduced maximum response of the α4β2V287L concentration-response relation is consistent with a reduction in receptors with the (α4)_3_(β2)_2_ stoichiometry. Also, the increased relative ACh response of the α5α4β2V287L-transfected cells is consistent with an α5-mediated increase in mutant receptor expression.

**Table 2:** Fitted parameters for α4β2, α4β2V287L, α5α4β2, and α5α4β2V287L ACh concentration-response relations in HEK cells using a membrane-potential dye.

Receptor	EC50 (μM)^a^	Hill coefficient	Maximum Response^b^	df^c^
α4β2	6 ± 1^d^	0.72 ± 0.06	0.95 ± 0.02	13
α4β2V287L	0.4 ± 0.1^#^^e^	0.7 ± 0.1	0.53 ± 0.02***^f^	13
α5α4β2	26 ± 5^#^	0.57 ± 0.04	1.06 ± 0.03	13
α5α4β2V287L	0.5 ± 1^#^	1.0 ± 0.3	0.67 ± 0.03***	13

^a^Concentration-response relations were fit to the Hill equation with three free parameters (EC50, Hill coefficient, maximum response).

α4β2V287L

^b^Mutant and α4β2 WT responses were normalized to the α4β2 WT value at 1 mM ACh. Similarly, α5α4β2V287L mutant and α5α4β2 WT responses were normalized to the α5α4β2 WT value at 1 mM ACh.

^c^Degrees of freedom (df) are the number of concentrations in concentration-response relation minus the three free parameters.

^d^Parameter values are mean ± S.E.

^e^(^#^) Significantly different from the α4β2 WT value at the 0.05 level.

^f^(***) Significantly different from the corresponding WT (α4β2, α5α4β2) value at the 0.001 level.

### β2V287L similarly affects the α4β2 ACh concentration-response relation in voltage-clamped N2a cells

**Fig 7:**
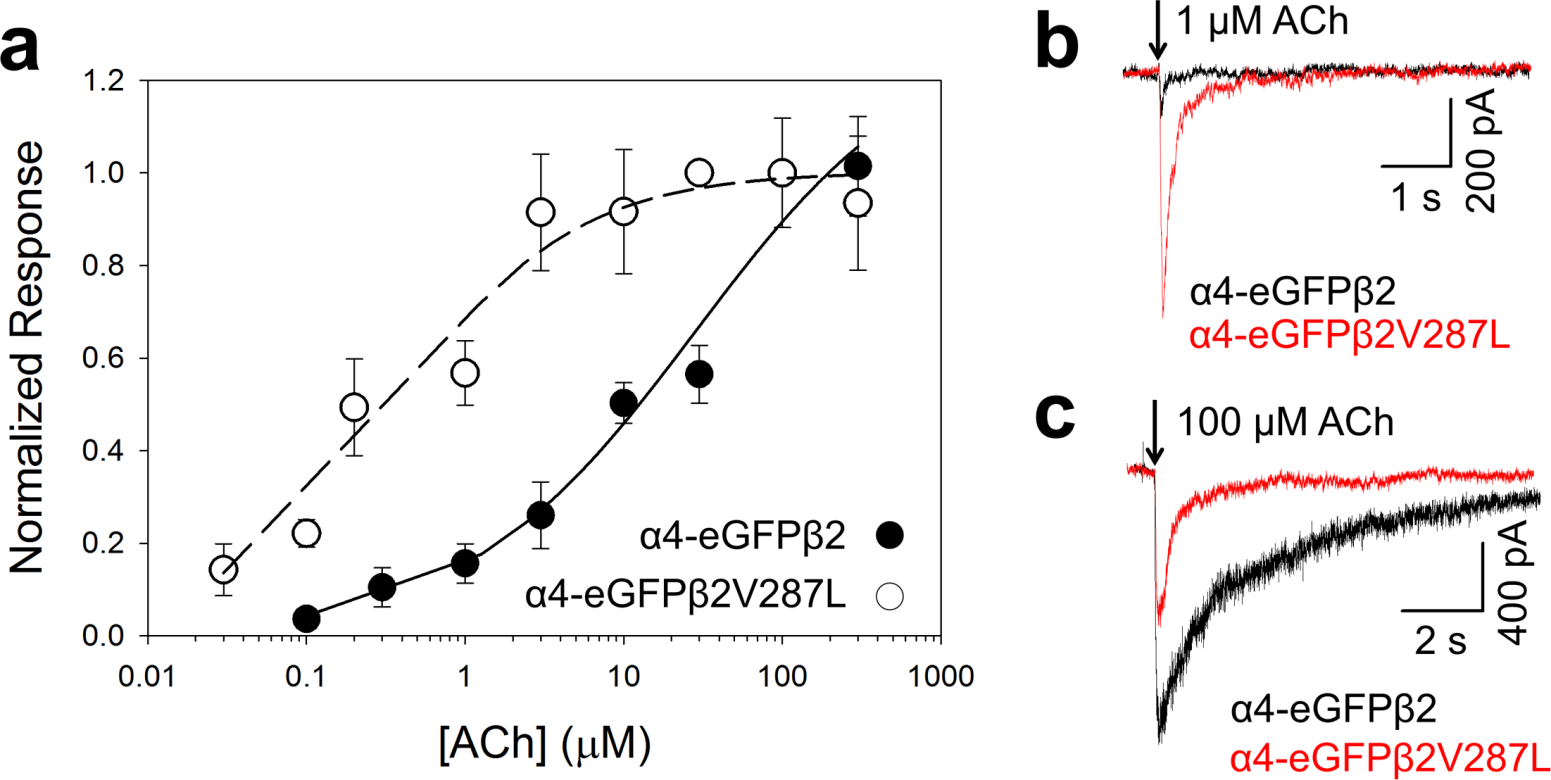
β2V287L increased the ACh sensitivity of α4β2 nAChRs in N2a cells but reduced maximum response. The α4 subunit was labeled with an enhanced green fluorescent protein (eGFP) tag to facilitate the identification of cells expressing α4β2 nAChRs. The cells were voltage-clamped at -60 mV in whole-cell mode. **a**. Normalized ACh concentration-response relations for the α4-eGFPβ2 (filled circles) or α4-eGFPβ2V287L receptors (open circles). The α4-eGFPβ2 and α4-eGFPβ2V287L ACh responses were normalized to the peak 100 μM response. The α4-eGFPβ2 symbol at 100 μM ACh is obscured by that for α4-eGFPβ2V287L. The dashed (α4-eGFPβ2V287L) and solid lines (α4-eGFPβ2) are fits to the three-parameter Hill equation (see text for values of fitted parameters). **b-c**. Superimposed traces of α4-eGFPβ2 (black) and α4-eGFPβ2V287L (red) responses to 300 ms applications of 1 (**b**) and 100 μM ACh (**c**). At 1 μM ACh, the α4-eGFPβ2V287L response was larger than α4-eGFPβ2 response. At 100 μM ACh, it was smaller. Downward arrows denote the onset of a 300 ms ACh application.

To further validate the fluorescent-dye data, we measured the effects of the β2V287L mutation on the ACh concentration-response relations of fluorescently tagged α4-eGFPβ2 nAChRs expressed in N2a cells using whole-cell patch clamping and rapid agonist microperfusion (to minimize desensitization) (Fig 7). Similar to the fluorescent-dye data (Fig 6), the voltage-clamp concentration-response data were fit to a three-parameter Hill equation (Eq 7). The β2V287L mutation shifted the α4-eGFPβ2 ACh concentration-response relation leftward (Fig 7A). The EC_50_ value for the α4-eGFPβ2 WT (30 ± 20 μM, n = 8 ACh concentrations) was 75 times larger than that for the α4-eGFPβ2V287L mutant (0.4 ± 0.1 μM, n = 9). The α4-eGFPβ2V287L (0.7 ± 0.2) and α4-eGFPβ2 Hill coefficients (0.6 ± 0.1) were both less than unity, suggesting receptor heterogeneity. The β2V287L mutation reduced the maximum ACh response but actually increased the magnitude of the response to 1 μM ACh (Fig 7B). The α4-eGFPβ2V287L response to 1 μM ACh (650 ± 230 pA, n = 9 cells) was significantly greater (*p* < 0.05) than the α4-eGFPβ2 value (100 ± 20 pA, n = 8) (Fig 7B). In contrast, the α4-eGFPβ2V287L response to 100 μM ACh (900 ± 240 pA, n = 9) was significantly (*p* < 0.05) less than the α4-eGFPβ2 value (1800 ± 400 pA, n = 8) (Fig 7C).

### β2V287L increases α5 surface expression

**Fig 8:**
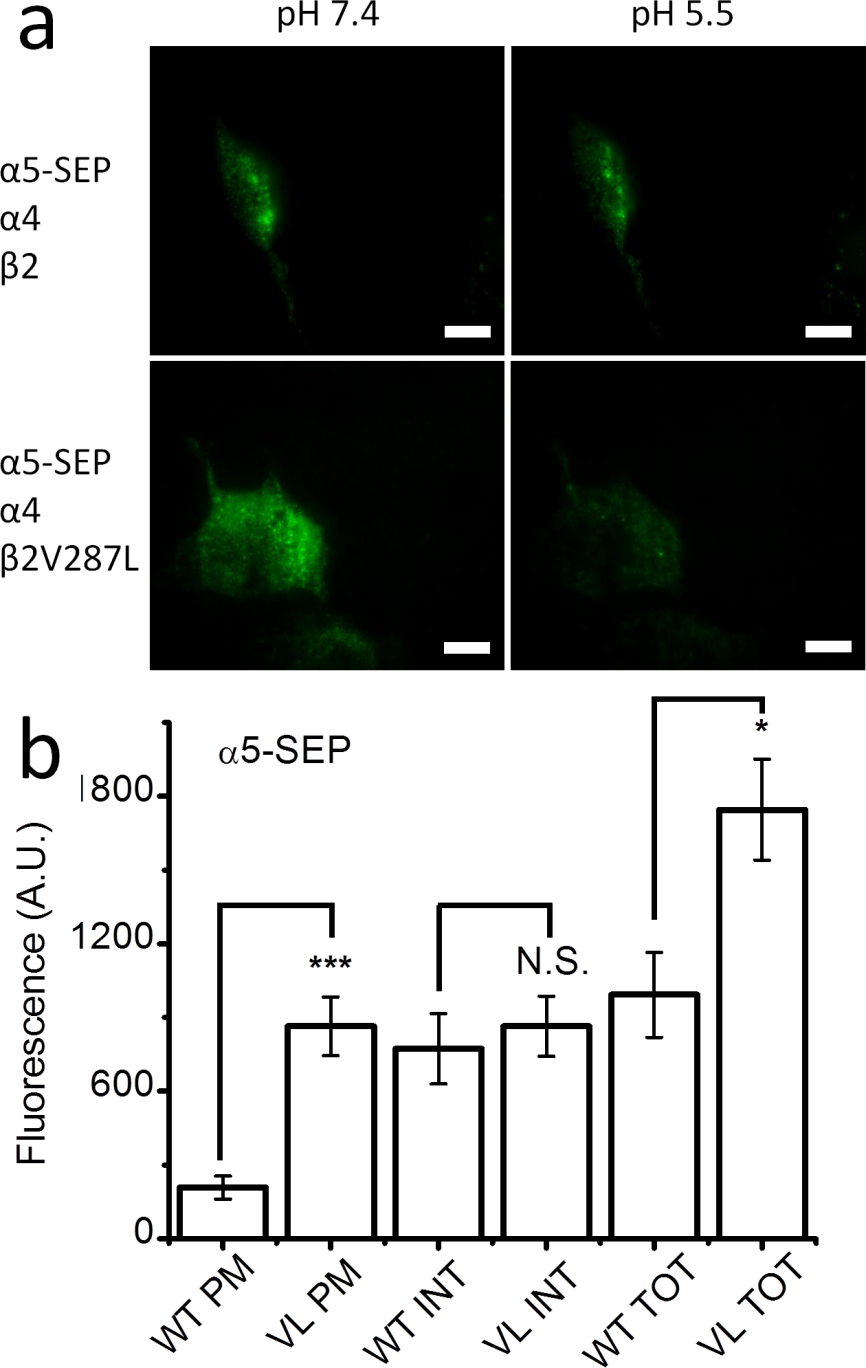
β2V287L increased α5 incorporation into surface α4β2* nAChRs. **a.** TIRF images of α5-SEP fluorescence in N2a cells co-transfected with α5-SEPα4β2 (top row), or α5-SEPα4β2V287L, cDNA (bottom row) at an extracellular pH of 7.4 (left column) and pH 5.5 (right column). **b**. Bar graphs of the PM, INT, and TOT fluorescent intensities of cells transfected with α5-SEPα4β2 (WT) or α5-SEPα4β2V287L (VL). The β2V287L mutation significantly (*p* < 0.001,***) increased TOT and PM α5-SEP fluorescence but did not affect INT fluorescence.

The membrane-potential-sensitive fluorescent dye experiments (Fig 5B) suggest that co-expression with α5 increases the surface expression of α4β2V287L nAChRs. To test this hypothesis further, we used SEP-tagged α5 subunits (α5-SEP) to compare the surface expression of α5-SEPα4β2 and α5-SEPα4β2V287L receptors. HEK cells were transfected with either α5-SEPα4β2 or α5-SEPα4β2V287L cDNA (α5:α4:β2 ratio of 1:1:1), and the total, internal, and PM α5-SEP fluorescence of the transfected cells were measured (Fig 8). Surprisingly, PM fluorescence of the mutant α5-SEPα4β2V287L receptor was nearly fourfold larger than that of the α5-SEPα4β2 (Fig 8B). There was also a small, but significant (*p* < 0.01), increase in the total α5-SEP fluorescence of the mutant receptors (Fig 8B). However, there was no significant difference in internal α5-SEP fluorescence (Fig 8B). Consequently, the mutant-induced increase in total fluorescence appears to be due mainly to a mutant-related increase in PM fluorescence, suggesting that β2V287L enhances the incorporation of α5 into surface α4β2* nAChRs. This effect may explain the α5-mediated increase in the relative mutant 300 μM ACh response detected in the fluorescent dye experiments above (Fig 5B). The detection of α5-SEP PM fluorescence in the α5-SEPα4β2-transfected cells also confirms that transfecting HEK cells with α5α4β2 cDNA in a 1:1:1 ratio actually results in the surface expression of α5α4β2 receptors.

## Discussion

Our major findings are that the β2V287L ADNFLE mutation increases the ACh sensitivities of both HS and LS α4β2 nAChRs, but reduces the proportion of LS α4β2 nAChRs expressed on the PM. Further, the mutation increases the surface expression of α5α4β2 nAChRs but does not alter their ACh sensitivity. The mutation shifts the combined (HS and LS) α4β2 ACh concentration-response relation leftward and reduces the maximum response. Despite this reduction in maximum response, β2V287L increases the voltage-clamped ACh response at 1 μM ACh, producing a net gain of function at this concentration. Previous experiments with knock-in mice show that β2V287L increases the ACh sensitivities of both HS and LS α4β2* nAChRs in brain synaptosomes and suppresses the functional expression of α4β2* LS receptors [18]. Our data show that these effects are replicated using heterologously expressed α4β2 nAChRs, suggesting that β2V287L inherently suppresses LS α4β2 nAChR expression regardless of the host cell type. Interestingly, the β2V287L mutation does not affect the ACh sensitivity of α5α4β2 nAChRs but it does dramatically increase the surface expression of this receptor subtype. Thus, the mutation produces a gain of function for α5α4β2 nAChRs throughout the effective ACh concentration range. All of these mutation-induced changes in the sensitivity and expression of α4β2 and α5α4β2 nAChRs could be potentially epileptogenic.

The use of SEP-tagged subunits is a novel approach for studying the effects of ADNFLE mutations on nAChR expression and subunit stoichiometry. The results of the SEP experiments confirm two important conclusions from the electrophysiological and fluorescent membrane-potential-sensitive dye experiments. The decrease in the maximum response and suppression of the LS component of ACh concentration-response relation suggest that β2V287L reduces the total number of α4β2 nAChRs expressed in the PM and alters subunit stoichiometry. However, mutant-induced changes in the single-channel conductance, agonist desensitization, and agonist efficacy of HS and LS mutant receptors could contribute to these effects. The SEP data confirm that β2V287L reduces (1) the total number of α4β2 nAChRs expressed on the PM and (2) the mean number of α4 subunits per receptor (which is consistent with a reduction in the proportion of LS receptors in the population).

Conversely, the agreement between the SEP and functional data strongly suggest that the effects of β2V287L mutation in the SEP experiments are not due to any particular interaction between the C-terminal SEP tag in the β2 subunit and the β2V287L mutation. SEP is basically a pH-sensitive eGFP protein. Previous results show that putting an SEP tag in the C-terminal region of α4 significantly reduces the α4β2 nAChR response, as opposed to putting eGFP in the M3-M4 intracellular loop of α4 [26]. Thus, adding an SEP tag to the C-terminal region of α4 may reduce α4β2 nAChR surface expression. However, the agreement between the effects of the β2V287L mutation on the PM expression of SEP-tagged receptors, and its effects on the ACh concentration-response relations of untagged α4β2 nAChRs (expressed in oocytes and HEK cells) and intracellularly tagged α4-eGFPβ2 nAChRs (expressed in N2a cells), suggest that our SEP results are not due to any particular interaction between the β2V287L mutation and C-terminal SEP tag. Consistent with our findings, previous PET data also show that the α4S284F and β2V287L ADNFLE mutations reduce nAChR density in the right dorsolateral prefrontal cortex of ADNFLE patients [14].

Finally, the SEP data show that β2V287L increases the number of α5 subunits in the PM of cells transfected with α5, α4, and β2 subunits, suggesting that the mutation significantly increases α5α4β2 nAChR surface expression. Previous data show that the α4S284F mutation also increases total α5α4β2 nAChR expression in oocytes (measured by [^3^H]epibatine binding after mAb 210 immunoprecipitation) [35]. However, the effect of the β2V287L mutation on α5α4β2 surface expression, measured with SEP, appears to be much larger.

Our α4β2 SEP data agree with the previously reported effects of the β2V287L mutation on ACh-induced ^86^Rb efflux from mouse brain synaptosomes [18]. The mutation significantly reduced total ACh-induced ^86^Rb efflux from cortical synaptosomes and, also the LS component of efflux more than the HS component [18]. In contrast, previous measurements of the effects of the β2V287L mutation on α4β2 nAChR stoichiometry using Fӧrster resonance energy transfer (FRET) between fluorescently tagged α4 and β2 subunits suggest that β2V287L increases the fraction of (α4)_3_(β2)_2_ LS receptors expressed in N2a cells [36]. However, unlike the present SEP experiments (which directly measure changes in PM expression), most of the FRET measured in these previous experiments probably comes from intracellular nAChRs in the ER. Thus, the ADNFLE mutations may increase the proportion of intracellular nAChRs with the (α4)_3_(β2)_2_ (LS) stoichiometry because they enhance intracellular retention of the LS α4β2 nAChRs. Another potential explanation is that FRET from partially assembled, intracellular receptors makes a significant contribution to the whole-cell FRET measurements, which confounds the effects of the mutations on the subunit stoichiometry of fully assembled receptors [37]. Regardless of the explanation, the agreement of the present electrophysiological and SEP data with previous synaptosomal ^86^Rb efflux data from β2V287L knock-in mice support the conclusion that β2V287L suppresses LS α4β2 surface expression.

Also consistent with a previous study of the α4S247F ADNFLE mutation [34], co-transfection of WT α4 and mutant β2V287L subunits with the WT α5 subunit improves mutant surface receptor expression, relative to a matching WT control. Hetero-pentameric nAChRs contain two αβ dimers and a fifth accessory subunit. The α4 subunit occupies the accessory subunit position in the LS α4β2 nAChR pentamer. However, in HS α4β2 and α5α4β2 nAChRs, β2 and α5 subunits occupy this position, respectively. Thus, β2V287-induced suppression of α4β2 LS expression suggests that the mutation destabilizes the PM expression of α4β2* nAChRs that have an α4 subunit in the accessory subunit position rather than a β2 or α5 subunit. Based on our results, the predicted *in vivo* effects of the β2V287L mutation are a loss of LS α4β2, and an increase in α5α4β2, nAChRs.

The suppression of surface LS α4β2 nAChR expression may account for two previously unexplained effects of the ADNFLE mutations. ADNFLE mutations reduce allosteric potentiation of the α4β2 ACh response by extracellular Ca^2+^ ions [15]. If Ca^2+^ potentiates the ACh response of HS α4β2 nAChRs less than that of LS nAChRs, then suppressing LS expression will reduce Ca^2+^ potentiation of the combined α4β2 ACh response, particularly in the upper portion of the effective ACh concentration range where the HS response dominates. Thus, a loss of LS α4β2 expression could potentially account for the diminished Ca^2+^ potentiation of the ADNFLE mutant ACh response. The α4S248F and α4(777ins3) ADNFLE mutations also increase the Hill coefficient of the α4β2 ACh concentration-response relation [38]. If these two mutations suppress LS α4β2 nAChR expression, then they could increase the Hill coefficient of the combined ACh concentration-response relation by increasing receptor homogeneity.

Despite strong evidence linking α4 and β2 nAChR subunit mutations to ADNFLE, there is no compelling evidence (such as block of the seizures by nicotinic antagonists) to suggest that the activation of mutant α4β2 nAChRs is the proximate cause of ADNFLE seizures. In fact, it appears more likely that the ADNFLE mutations generate spontaneous seizures by persistently altering brain circuitry during development [17]. Nicotinic receptors are present in the human brain at birth but ADNFLE seizures typically do not begin until 5–15 years of age [7]. This period coincides with a developmental shift in the focus of maximum SWA from the occipital to frontal lobe during sleep [9]. The mean age of onset for patients with the α4S247F mutation is 11.7 years (median 8 years) and the seizures typically persist through adult life [7]. Maximum SWA power in the frontal lobe undergoes a step-like increase at ages 8–11 that persists into adult life [9]. Because SWA represents an increase in the synchronized firing of cortical neurons during sleep, this developmental shift may facilitate the initiation of ADNFLE seizures in the frontal lobe. Previous experiments with mice conditionally-expressing a β2V287L transgene suggest that there is a critical period for ADNFLE seizure development [17]. Silencing the β2V287L mutant transgene during early development prevents adult seizures despite expression of the mutant transgene in the adult mouse [17]. Moreover, inactivating the mutant transgene in adult mice does not prevent seizure activity. Thus, transgene expression in the adult mouse is insufficient for seizure expression.

Previous studies of β2* nAChRs show that they play a role in the development of cortical excitatory synaptic transmission [39, 40]. Genetic deletion of the β2 subunit reduces dendritic spine density in pyramidal neurons in the prelimbic/infralimbic area [39] and causes a redistribution of glutamatergic synapses from dendritic spines to the dendritic shaft in pyramidal neurons [40]. In contrast, the activation of β2* nAChRs by nicotine during early development increases spine formation in the dendrites of pyramidal neurons [40]. Our results show that β2V287L increases the response of α4β2 nAChRs expressed in HEK cells to an ACh concentration (1 μM) near the foot of the concentration-response relation, even though it reduces the maximum ACh response. Thus, the increased mutant β2* nAChR response to sub-saturating ACh concentrations could enhance seizure susceptibility by altering the course of normal development for excitatory synaptic transmission in the brain.

Nicotinic receptors containing α5 subunits (α5* nAChRs) likewise contribute to cortical development [41]. Co-expressing α5 with α4 and β2 nicotinic subunits increases the Ca^2+^ permeability of the expressed nAChRs [42] and calcium is an important intracellular messenger. Corticothalamic neurons in layer 6 of the prefrontal cortex express α5α4β2* nAChRs [43]. These receptors appear to be postsynaptic and regulate short-term plasticity at layer 6 nicotinic synapses [44]. They also play an important role in pruning back the apical dendritic tree of layer 6 pyramidal neurons during development [45]. At birth, the apical dendrites of most layer 6 pyramidal neurons extend to layer 1. During development, most of these dendrites retract from layer 1. However, genetically deleting the α5 subunit largely prevents this retraction [45]. A β2V287L-mediated increase in α5α4β2* nAChR expression could have the opposite effect and actually enhance dendritic retraction in these neurons. Genetic deletion of the α5 subunit also reduces the sensitivity of mice to nicotine-induced seizures [46, 47]. Thus, increasing α5α4β2* nAChR expression may increase seizure susceptibility. Previous studies show that β2V287L knock-in mice display an enhanced sensitivity to nicotine-induced seizure behaviors [18, 19].

Because of the rarity of ADNFLE mutations, human patients are heterozygous for the mutations. Thus, β2* nAChRs in these patients may contain a mixture of WT and mutant β2 subunits. We did not attempt to address this issue in the present study. Co-expressing WT and mutant β2 subunits in our experiments would make interpreting the data nearly impossible. The only way to adequately address this issue is to construct concatameric receptors containing WT and mutant β2 subunits and, these experiments are clearly beyond the scope of the present study. Nevertheless, a comparison of the amplitude of the LS component for ACh-induced ^86^Rb release from cortical synaptosomes in heterozygous and homozygous β2V287L knock-in mice suggests that a significant reduction in the LS component is still present in the heterozygous mice and that this reduction is about half that of the homozygous mutant mice [18]. The formation of LS α4β2 nAChRs requires the insertion of an α4, rather than a β2, subunit at the accessory position in the pentamer. Assuming that α4 and β2 subunits compete for insertion at this site, the straightforward explanation for the intermediate reduction in LS α4β2 expression in the heterozygous β2V287L knock-in mice is that the β2V287L mutation increases the probability that a β2 subunit is inserted at that site in proportion to its relative abundance in the cell. Thus, the presence of the β2V287L mutant subunits diminishes the probability that α4 is inserted at that site. Following similar reasoning, we expect the β2V287L-mediated increase in α5α4β2* nAChR expression in the heterozygote to be half of that in the homozygote.

How might suppression of LS α4β2 nAChR expression on the PM contribute to ADNFLE epileptogenesis? A recent study suggests that the co-expression of HS and LS α4β2 nAChRs in a presynaptic nerve terminal enhances the dynamic range of the presynaptic nicotinic response [30]. In the cortex, presynaptic nAChRs on the terminals of cholinergic neurons act as positive autoreceptors that increase ACh release [48, 49]. In the absence of LS receptors, the response of these receptors may saturate at lower levels of ACh release and reduce the activation of postsynaptic muscarinic receptors. Stimulation of the basal forebrain desynchronizes the EEG in anesthetized animals by activating postsynaptic, cortical muscarinic receptors and shifts pyramidal neuron firing from the bursting, to tonic, mode [50]. The loss of LS α4β2 nAChRs could limit the ability of ACh release to dampen EEG synchronization during periods of intense cortical activity.

In conclusion, our results show that the β2V287L ADNFLE mutation increases the ACh sensitivities of both HS and LS α4β2 nAChRs (but not that of α5α4β2 nAChRs). Moreover, it suppresses surface LS α4β2 expression and increases α5α4β2 expression. The suppression of LS α4β2 expression may be a common feature of the ADNFLE mutations and could potentially explain their previously reported effects on allosteric Ca^2+^ potentiation of the ACh response. Increased α4β2 ACh sensitivity, suppressed LS α4β2 expression, and increased α5α4β2 expression may contribute to ADNFLE ictogenesis by (1) altering the development of excitatory synaptic connections in the brain and/or (2) impairing the ability of presynaptic nAChR autoreceptors to properly regulate cortical ACh release.
